# Transcranial direct current stimulation for memory enhancement: from clinical research to animal models

**DOI:** 10.3389/fnsys.2014.00159

**Published:** 2014-09-04

**Authors:** Djamila Bennabi, Solène Pedron, Emmanuel Haffen, Julie Monnin, Yvan Peterschmitt, Vincent Van Waes

**Affiliations:** ^1^EA 481 Laboratory of Integrative and Clinical Neuroscience, University of Franche-Comté/SFR FED 4234Besançon, France; ^2^INSERM CIC-IT 808 Clinical Investigation Centre for Innovative Technology, University Hospital of BesançonBesançon, France

**Keywords:** tDCS, neuromodulation, prefrontal cortex, cognitive enhancement, memory

## Abstract

There is a growing demand for new brain-enhancing technologies to improve mental performance, both for patients with cognitive disorders and for healthy individuals. Transcranial direct current stimulation (tDCS) is a non-invasive, painless, and easy to use neuromodulatory technique that can improve performance on a variety of cognitive tasks in humans despite its exact mode of action remains unclear. We have conducted a mini-review of the literature to first briefly summarize the growing amount of data from clinical trials assessing the efficacy of tDCS, focusing exclusively on learning and memory performances in healthy human subjects and in patients with depression, schizophrenia, and other neurological disorders. We then discuss these findings in the context of the strikingly few studies resulting from animal research. Finally, we highlight future directions and limitations in this field and emphasize the need to develop translational studies to better understand how tDCS improves memory, a necessary condition before it can be used as a therapeutic tool.

Non-invasive neuromodulatory techniques including tDCS have been shown to improve performance on a variety of cognitive domains. tDCS is a painless stimulation method that delivers subthreshold electrical currents to the brain and manipulates the resting membrane potential of cortical neurons (e.g., Stagg and Nitsche, 2011). Since the dorsolateral prefrontal cortex (DLPFC) is a crucial element in the neural network subserving executive functions (i.e., working memory, planning, goal-oriented behavior, attention, and inhibitory control; Wood and Grafman, 2003), targeting this area with neuromodulatory techniques represents a promising therapeutic option for improving cognition. In this mini-review, we summarize data obtained from clinical trials (see Tables 1A, B) and animal models focusing on tDCS-induced memory enhancement in healthy subjects and in subjects with psychiatric and neurological disorders known to induce mild to severe cognition impairments. Limitations and perspectives are then discussed.

**Table 1A:** **Studies investigating the cognitive effects of tDCS in healthy subjects**.

**Study**					**tDCS parameters**				**Results**
**Author**	**Design**	**n**	**Task**		**A/C**	**I (mA)**	**D (min)**	**E (cm^2^)**	
Fregni et al., 2005	Cross over	15	n-back task	Online	F3/FP2	1	10	35	Improvement in accuracy (more correct responses)
	Single blind								No improvement in reaction time
Ohn et al., 2008	Cross over	15	n-back task	Online	F3/FP2	1	20	35	Increased number of correct responses after 30 min of stimulation with anodal stimulation
	Single blind			Offline					
Lally et al., 2013	Cross over	21	n-back task	Online	F3/cheek	1	10	35	Improvement of performance during the first stimulation phase with active stimulation
	Double blind			Offline					
Mulquiney et al., 2011	Cross over	10	Cogstate	Online	F3/FP2	1	10	35	2-back task: no effects of session or time in accuracy; improvement in reaction time Sternberg task: no effect of session or time
			Sternberg task	Offline					
Marshall et al., 2005	Cross over	12	Modified	Online	F3/F4	0.26	15	64	No improvement in accuracy
	Double blind		Sternberg task						Slower reaction time after anodal and cathodal tDCS
Andrews et al., 2011	Cross over	10	n-back task	Online	F3/FP2	1	10	35	Previous application of tDCS during the n-back task resulted in increased performance on digit span forward
			Digit span tasks	Offline					
Berryhill and Jones, 2012	Cross over	25	n-back task	Offline	F3/cheek	1.5	10	35	Low education group: unchanged or impaired performance
					F4/cheek				High education group: improved performance
Teo et al., 2011	Cross over	12	n-back task	Online	F3/FP2	1 or 2	20	35	n-back task: decrease reaction time during the last 5 min of 2 mA session.
	Double blind		Sternberg task	Offline					Sternberg task: no difference in reaction time and accuracy between 1 mA, 2 mA, or sham stimulation
Gladwin et al., 2012a	Cross over	14	Sternberg task	Online	F3/FP2	1	10	35	Improvement in reaction time, influence of interference
				Offline					
Gladwin et al., 2012b	Cross over	20	Modified version of the IAT	Offline	F3/FP2	1	10	35	Improvement in reaction time in the congruent rather than in the incongruent condition
Kincses et al., 2004	Cross over	14	PCL	Online	F3/Cz	1	10	35	Improvement of implicit learning by anodal but not cathodal stimulation
Hammer et al., 2011	Cross over	36	Recognition memory task	Online	F3/FP2	1	30	35	Cathodal stimulation hampered memory performance after errorful learning, whereas anodal stimulation did not alter encoding and memory retrieval
	Single blind			Offline					
Manenti et al., 2013	Cross over	64	Episodic memory task	Online	F3/F4	1.5	6	35	Improvement of verbal episodic memory with anodal tDCS applied during the retrieval phase Better performances in young subjects
	Single blind								
Zwissler et al., 2014	Cross over	85	Episodic memory task	Online	F3/con-tralateral musculus deltoideus	1	15	35	Anodal tDCS increased whereas cathodal stimulation decreased the number of false alarms to lure pictures in subsequent recognition memory testing
	Double blind								

*Cz, midline central (international 10/20 EEG system); F3, left dorsolateral prefrontal cortex; F4, right dorsolateral prefrontal cortex; FP2, supraorbital right; IAT, Implicit Association Test; PCL, probabilistic classification learning; I, intensity; D, duration; E, electrodes size*.

**Table 1B:** **Studies investigating the cognitive effects of tDCS in psychiatric diseases**.

**Study**					**tDCS parameters**				**Results**
**Author**	**Design**	**n**	**Task**		**A/C**	**I (mA)**	**D (min)**	**E (cm^2^)**	
**DEPRESSION**									
Brunoni et al., 2013	Double blind RCT	28 UP	Probabilistic classification learning	Online	F3/F4	2	30	25	No improvement in implicit learning after real stimulation
Fregni et al., 2006	Double blind RCT	18 UP	Digit span forward and backward test	Online	F3/FP2	1	10	35	Improvement in working memory
Oliveira et al., 2013	Double blind RCT	28 UP	n-back task	Online	F3/F4	2	30	25	Enhancement of working memory
									Increase rate of correct responses
									Increase ability to discriminate between correct responses and false alarms
Wolkenstein and Plewnia, 2013	Double blind RCT	22 MDD	Delayed- response working memory task	Online	F3/Right upper arm	1	20	35	Enhancement of working memory performance and elimination of attentional bias
Ferrucci et al., 2009	Open label	8 MDD	Sternberg Task Word recognition task	Offline	F3/F4	2	20	32	Cognitive tasks showed no significant difference between active or sham stimulation
			Posner paradigm						
Loo et al., 2012	Double blind RCT	64 MDD	RAVLT, Stroop Test, COWAT, Digit span, SDMT	Offline	F3/F8	2	20	35	Improvement of working memory performances, indexed by the SDMT, after 1 tDCS session
									No improvement in cognitive performances after 15 sessions
Palm et al., 2012	Double blind RCT	22 MDD	VLMT, RWT LNS_WAIS_	Offline	F3/FP2	1 or 2	20	35	Cognitive tasks showed no significant difference between active or sham stimulation
**SCHIZOPHRENIA**									
Vercammen et al., 2011	Single blind	20	Probabilistic classification learning	Online	F3/FP2	2	20	35	Improvement in implicit learning after real stimulation in a subset of patient
	Cross over								
Hoy et al., 2014	Double blind RCT	18	nback	Offline	F3/FP2	1 or 2	20		Improvement in working memory at 2 mA
Goder et al., 2013	Cross over	14	RAVLT	Offline	F3/F4	0–0.3	During sleep	64	Improvement in working memory
**ALZHEIMER**									
Boggio et al., 2009	Double blind RCT	10	Digit span test Visual recognition	Online	F3/ FP2	2	30	35	Improvement in working memory after prefrontal and temporal stimulation
			Memory task Stroop test		T7/FP2				No effect on digit span and Stroop performance
Cotelli et al., 2014	Double blind RCT	36 (mild to moderate)	Face-name association memory task	Offline	F3/Right deltoid muscle	2	24		No additive effects of anodal tDCS on memory performance when combined with memory training
			Memory training						
**PARKINSON**									
Boggio et al., 2006	Double blind RCT	18	n-back task	Online	F3/ FP2	1 or 2	20	35	Improvement in accuracy No improvement in reaction time
									No effect at 1 mA
Pereira et al., 2013	Cross over	16	Semantic fluency task phonemic task	Offline	F3/FP2	2	20	35	Improvement in the phonemic fluency task after DLPFC tDCS
					P3-T5/FP2				
**POST-STROKE**									
Kang et al., 2009	Double blind RCT	10	Go/No-Go	Offline	F3/FP2	2	20	25	Improvement in response accuracy at 1 and 3 h post-stimulation
Jo et al., 2009	Double blind RCT	10	n-back task	Offline	F3/FP2	2	30	35	Improvement in the two-back task after DLPFC tDCS
Park et al., 2013	Double blind RCT	11	Seoul computerized neuropsychological test	Offline	F3/F4	2	30	25	Improvement in attention when combined with cognitive rehabilitation

*COWAT, Controlled Oral Word Association Test; F3, left dorsolateral prefrontal cortex (international 10/20 EEG system); F4, right dorsolateral prefrontal cortex; FP2, supraorbital right; F8, lateral aspect of the right orbit; LNS_WAIS_, Letter Number Sequencing Task of the Wechsler Adult Intelligence Scale; MDD, Major Depressive Disorder; RAVLT, Ray Auditory Verbal Learning Test; RCT, Randomized Controlled Trial; RWT, Regensburg Word Fluency Test; SDMT, Symbol Digit Modalities Test; UP, Unipolar Depression; I, intensity; D, duration; E, electrodes size; VLMT, Verbal Learning Memory Test, P3-T5, left temporo-parietal cortex*.

## Human studies

### tDCS for cognitive enhancement in healthy subjects

To date, most studies conducted in healthy individuals have assessed the effect of tDCS in enhancing verbal and visuospatial components of working memory (WM) and learning processes. Fregni et al. found that online anodal tDCS at 1 mA applied over the left DLPFC enhances accuracy in a three-back letter task compared with cathodal stimulation of the same area or anodal stimulation of the primary motor cortex (Fregni et al., 2005). Based on the same paradigm, Ohn et al. investigated the time-dependency of tDCS and found an increased number of correct responses starting 20 min after the beginning of active stimulation compared to sham, whereas earlier measurements did not reveal any stimulation effects (Ohn et al., 2008). More recently, Lally et al. confirmed these results in a larger cohort, but only when subjects were tested during the stimulation session (online), without a persisting effect 48 h later (Lally et al., 2013). Mulquiney et al. obtained discordant results in a sample of 10 healthy volunteers, with no improvement in accuracy but in speed performance after anodal tDCS (Mulquiney et al., 2011). However, Marshall et al. reported increased reaction time in the same task during both anodal and cathodal bilateral intermittent stimulation over the DLPFC (Marshall et al., 2005). Andrews and collaborators investigated the impact of 1 session of anodal tDCS delivered during a WM task (n-back task) on performances on a subsequent WM task (digit span forward) (Andrews et al., 2011). Upon completion of the n-back task, they observed a significant improvement in performance on the digit span forward task. Berryhill and Jones enhanced WM by application of anodal tDCS over the left or right DLPFC in subjects with a high educational level, whereas tDCS provided no benefit in WM performance to a less educated group (Berryhill and Jones, 2012). Interestingly, Teo et al. found that WM performances are influenced by current strength of anodal tDCS (Teo et al., 2011). Gladwin et al. explored the impact of anodal left DLPFC tDCS on Sternberg task completion when distractor stimuli were presented during the retention period. tDCS improved reaction time only when the incorrect choice had been a distractor suggesting stimulation might have an effect on selective attention. In a subsequent study, they showed that tDCS improves reaction time in an implicit association test without affecting the subjects' ability to overcome bias (Gladwin et al., 2012a,b).

In addition, tDCS has been recently used as an investigative tool in other memory domains. With regard to implicit memory (probabilistic classification learning), Kincses et al. first demonstrated that anodal tDCS performed over the left DLPFC at 1 mA in healthy volunteers resulted in immediate improvement in implicit learning (Kincses et al., 2004). Hammer et al. showed that cathodal stimulation hampered memory performance after errorful learning, whereas anodal stimulation did not alter encoding and memory retrieval (Hammer et al., 2011). Manenti et al. found that anodal stimulation enhances the long-term episodic memory capacities of young and older subjects with more robust effects in young participants (Manenti et al., 2013). Plewnia and collaborators also reported that tDCS shapes accuracy of episodic memory via polaritiy-specific modulation of false recognition. When applied during encoding of pictures, anodal tDCS increased whereas cathodal stimulation reduced the number of false alarms (i.e., responses to highly similar distracter images) in subsequent recognition memory testing (Zwissler et al., 2014).

### tDCS for cognitive enhancement in psychiatric and neurological disorders

Bifrontal tDCS has been shown to prevent procedural learning in depressive states, possibly by inducing a decrease in the activity of the right DLPFC (Brunoni et al., 2013). Beneficial effects of online stimulation applied over the left DLPFC have been reported for working memory, attentional performances, and information processing in depressed patients (Fregni et al., 2006; Oliveira et al., 2013; Wolkenstein and Plewnia, 2013). However, two randomized controlled trials and one open-label trial failed to replicate this finding with offline stimulation, suggesting that multiple tDCS sessions do not have cumulative cognitive enhancing effects (Ferrucci et al., 2009; Loo et al., 2012; Palm et al., 2012).

Only a small number of studies have examined the impact of tDCS on selective cognitive domains altered in schizophrenia. Focusing on working memory, Vercammen et al. reported that a single session of anodal tDCS to the left DLPCF improves probabilistic association learning in a specific subset of schizophrenic patients (Vercammen et al., 2011). These findings were interpreted as an enhancement of DLPFC function primarily in individuals with relatively higher neural and cognitive reserve. Hoy et al. observed the same tDCS effects on a working memory task after a 2 mA stimulation (Hoy et al., 2014). Göder et al. showed improved sleep-associated memory consolidation in patients with schizophrenia when anodal tDCS oscillating at a frequency of 0.75 Hz was applied during sleep (Goder et al., 2013).

Cognitive enhancing properties of tDCS have also been explored in a number of neurological diseases. For example, in Alzheimer disease, Boggio et al. (2009) reported short-term facilitation effects on visual recognition memory after prefrontal and temporal anodal tDCS applied 30 min at 2 mA, with no changes in attention. More recently, Cotelli et al. demonstrated that repeated sessions of anodal tDCS to the left DLFPC plus computerized memory training led to an increase in performance in a face-name association task (Cotelli et al., 2014). However, combined treatment failed to ameliorate the memory performance more than memory training alone suggesting an absence of effects of tDCS in this paradigm. It has also been shown that a single tDCS session can ameliorate memory deficits in Parkinson's disease. Boggio et al. enhanced WM by application of anodal tDCS over the left DPFC at 2 mA, whereas stimulation with intensities of 1 mA or of other area (motor cortex) provided no benefit in WM performance (Boggio et al., 2006). Pereira et al. found that anodal tDCS (at 2 mA) applied over the left DLPFC enhanced performance and functional connectivity in task-related networks in a verbal fluency task tested during fMRI (Pereira et al., 2013).

Kang et al. reported increased response accuracy in a Go/NoGo task tested 1 and 3 h after anodal stimulation at 2 mA over the DLPFC in 10 patients with post-stroke cognitive decline (MMSE ≤ 25) (Kang et al., 2009). Jo et al. also reported that 10 patients with subacute stroke achieved a significant improvement in the accuracy of verbal two-back working memory after receiving the tDCS to the left prefrontal cortex at an intensity of 2 mA for 30 min (Jo et al., 2009). Park et al. found that the concomitant use of anodal tDCS with a computer-assisted cognitive rehabilitation program had a significant effect on improving attention in post-stroke patients with mild-to-moderate cognitive dysfunction (Park et al., 2013).

In spite of the increasing number of clinical studies showing beneficial effects of prefrontal tDCS on the domains of learning and memory, its mechanism of action remains unclear. Recent clinical studies have started to tackle this question (e.g., Keeser et al., 2011a,b; Amadi et al., 2013; Dayan et al., 2013; Palm et al., 2013; Plewnia et al., 2013; Stagg and Johansen-Berg, 2013); however, the cellular mechanisms underlying tDCS will likely require the use of animal models.

## Animal models of tDCS

Animal models provide a powerful tool to identify the mechanisms by which tDCS modulates neural network function to support improved cognition. In rats, tDCS was first used to evaluate the safety limits of cathodal stimulations (Liebetanz et al., 2009) and to map brain activation patterns after tDCS (Takano et al., 2011). In the latter study, the authors observed significantly increased fMRI signal intensities in the frontal cortex and nucleus accumbens of rats after anodal tDCS (of the frontal lobe), suggesting that tDCS induces neuronal activation both in cortical and subcortical areas. To date, few animal studies have addressed the impact of tDCS on learning and memory processes.

### tDCS for cognitive enhancement in healthy animals

Similar to humans, the prefrontal cortex (or more generally speaking, the frontal lobe) has been the main target of animal studies for its implication in working memory. In a recent paper, Dockery et al. performed experiments in rats using the Allothetic Place Avoidance Alternation Task (APAAT), a behavioral model of visuospatial working memory and skill learning (Dockery et al., 2011). In this paradigm, a recent memory is engaged by the necessity to remember the location of a to-be-avoided sector (punished by an electric shock), which is alternated daily. tDCS on the frontal lobe (30 min/day before the APAAT task [3 days in total], 200 μA, epicranial electrode: 3.5 mm^2^ over the frontal lobe, counter electrode: 10.5 cm^2^ placed between the shoulders) had no measurable short-term effect on on-going place avoidance learning. However, in a follow-up session (18 days after the last APAAT session), the rats previously treated with cathodal (but not anodal) tDCS showed significantly more efficient place avoidance and skill retention compared to controls. Other types of memory, such as associative learning processes, can also be affected by tDCS (Marquez-Ruiz et al., 2012). In this case, tDCS was applied to behaving rabbits *via* four silver-ball stimulating electrodes (1 mm in diameter, placed symmetrically above the skull 3 mm from the right S1 vibrissa area on the somatosensory cortex) with a saline-soaked sponge (35 cm^2^ surface area) attached to the contralateral ear serving as the counter electrode. The authors found that the acquisition of classical eyeblink conditioning is potentiated or depressed by the simultaneous application of anodal or cathodal tDCS, respectively, when stimulation of the whisker pad was used as a conditioned stimulus. These results suggest that tDCS modulates the sensory perception processes necessary for this type of associative learning (Marquez-Ruiz et al., 2012).

**Figure 1:**
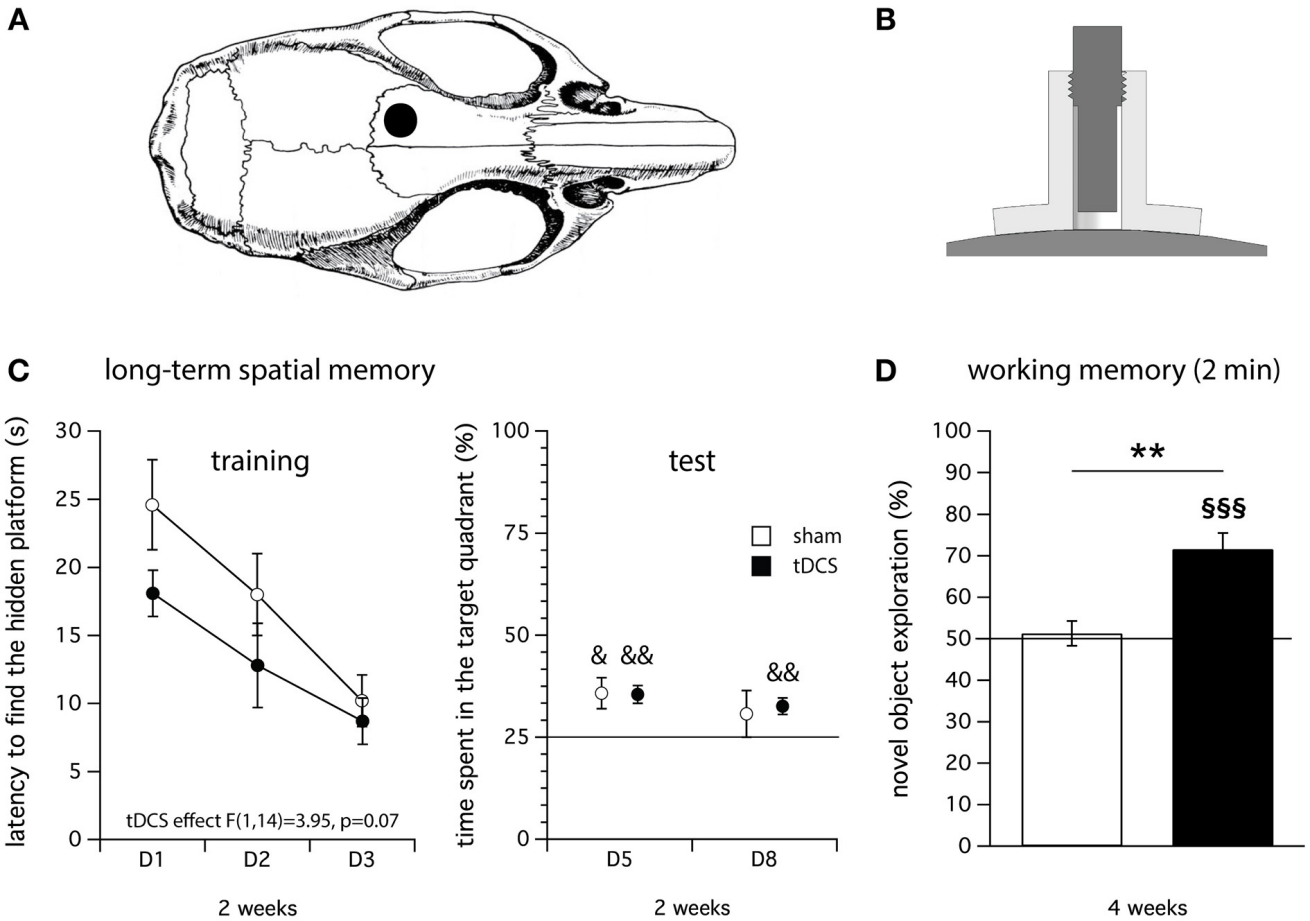
**Illustration of the tDCS device used to deliver the electrical stimulation in mice (Pedron et al., 2014) and main results obtained on cognition. (A)** The center of the stimulation electrode (anode) is positioned over the left frontal cortex 1 mm anterior to the coronal fissure and 1 mm left of the sagittal fissure (Paxinos and Franklin, 2001). The cathode (rubber-plate electrode, 9.5 cm^2^) is placed onto the ventral thorax (not shown). **(B)** Anode: A tubular plastic jacket (internal diameter: 2.1 mm) is surgically fixed onto the skull and filled with saline solution before the stimulation. The stimulation electrode is screwed into the tubular plastic jacket and immersed in the saline solution. Only the saline solution is in contact with the skull. **(C)** Four month-old Swiss female mice were subjected to repeated anodal tDCS for 5 consecutive days (2 × 20 min/day constant current, 0.2 mA). Long-term spatial memory was evaluated 2 weeks after the last stimulation in the Morris water navigation task (training: left; test: right). tDCS significantly improved long-term spatial memory. **(D)** Effect of repeated anodal tDCS on working memory evaluated in an object recognition task (inter-trial interval: 2 min) 4 weeks after the last stimulation. The novel object exploration (score in %) was significantly higher than 50% in the tDCS group, reflecting a better working memory performance compared to sham group for which the score was not significantly different than 50%. ^&^*p* < 0.05 and ^&&^*p* < 0.01 vs. 25%, ^**^p < 0.01 vs. sham, ^§§§^*p* < 0.001 vs. 50%; *N* = 8 per group.

Recently, we have adapted a model of tDCS in mice and tested its validity in a wide range of behavioral paradigms (Pedron et al., 2014). We applied repeated anodal tDCS over the left frontal cortex of the mouse (see, Figures 1A,B) and used a 2 × 20 min/day stimulation paradigm at 200 μA for 5 consecutive days. In agreement with human studies, our data suggest that repeated anodal tDCS improves long-term spatial memory (in the Morris water maze, Figure 1C) and working memory (object recognition, Figure 1D) without affecting behaviors such as locomotor activity and anxiety-related behaviors (Pedron et al., 2014).

Finally, Marshall and collaborators have investigated the interaction of tDCS with hippocampo-neocortical rhythms and reported that a transcranial slow oscillation stimulation during sleep enhances memory consolidation in rats (anodes: bilaterally over the prefrontal cortex; return electrodes: over the cerebellum; sinusoidal constant current fluctuating between 0 and 5.6 μ A at a frequency of 1.5 Hz applied during non-rapid eyes movement sleep) (Binder et al., 2014a,b).

### Cognitive enhancement in animal models of neurological disorders

To the best of our knowledge, tDCS has yet to be tested for enhancing cognition in animal models of psychiatric disorders, but it has been shown to facilitate recovery from cognitive impairments induced by stroke or status epilepticus in rats. After cerebral ischemia, Yoon et al. employed a cup-shaped anodal stimulation electrode positioned at the ischemic borderline, and a rectangular rubber cathodal electrode (80 × 60 mm) fixed on the anterior chest (Yoon et al., 2012) to inject a direct current at an intensity of 200 μA for 20 min, once a day for 5 consecutive days. Both early (1 day) and late (1 week after ischemic injury) treatment had a beneficial outcome on cognition (spatial memory evaluated in the Barnes maze test) without exacerbating ischemic volume. Interestingly, this effect was not present 1 day after tDCS, but began to appear 2 weeks after the stimulations and was maximal after 4 weeks. Therapeutic effects of tDCS on cognition were associated with an increase in the expression of Map-2 (a stabilizer of microtubules growth) and Gap-43 (a neuronal growth-promoting gene) in the early treatment group and in the late treatment group, respectively, in both peri-lesional and contra-lesional cortex. Kamida et al. used cathodal tDCS (1.5 mm to the right, 2 mm anterior from bregma; counter electrode: 1 cm needle electrode inserted into the back of the neck, 30 min per day for 2 weeks at 200 μA) to evaluate its effect on seizures and spatial memory deficits following pilocarpine-induced status epilepticus in immature rats (Kamida et al., 2011). Repeated cathodal tDCS reduced seizures, spatial memory impairments, status epilepticus-induced hippocampal cell loss, and supragranular and CA3 mossy fiber sprouting.

## Potential mechanisms of action and perspectives

### Candidate mechanisms underlying tDCS action on cognition

To date, it is known that tDCS modifies the resting membrane potential when online and induces prolonged offline after-effects similar to long-term potentiation/depression (Paulus, 2004), considered to be the cellular mechanisms of learning and memory. For example, in humans the long-lasting effects of tDCS (both anodal and cathodal) on the primary motor cortex are suppressed after NMDA-receptor blockade indicating a dependence on glutamatergic activity (Liebetanz et al., 2002). Moreover, previous experiments in rats have shown that anodal polarization directly applied to the cortex has the ability to modulate neural plasticity (i.e., c-Fos activation) *via* activation of NMDA receptors (Islam et al., 1995).

Our team has started to investigate the role of adult neurogenesis as a mechanism involved in tDCS action. Neurogenesis in the hippocampus is of particular interest as tDCS induces both antidepressant effects and enhances cognition in humans and mice, two phenomena critically linked to the generation of new neurons in the adult dentate gyrus (Deng et al., 2010; Eisch and Petrik, 2012). In addition, the time course for the onset of long term tDCS effect on depression-related behavior and on cognition in our animal model (after several weeks, Pedron et al., 2014) is consistent with the delay necessary for newly generated cells in the hippocampus to be functionally integrated (Klempin et al., 2010). Of particular interest is the impact of tDCS on brain-derived neurotrophic factor (BDNF) levels. This growth factor is important for long-term memory (Bekinschtein et al., 2008), is involved in depressive-like behaviors and antidepressant drug action, and can modulate neurogenesis levels (Castren and Rantamaki, 2010; Vithlani et al., 2013). A recent study has shown that BDNF activation is necessary for DCS-induced long-term potentiation in mouse M1 slices (Fritsch et al., 2010). Enhancement of motor skill acquisition by anodal tDCS also seems to be related to BDNF, as the BDNF val66met polymorphism in humans is associated with decreased proclivity to tDCS-induced benefits on skill learning (Fritsch et al., 2010). Other indirect mechanisms cannot be ruled out, such as the impact of tDCS on cortical blood perfusion (Wachter et al., 2011; Stagg et al., 2013).

### Limitations and future directions

One outstanding question in the above-mentioned studies is: where does the current flow? Considering that tDCS has poor spatial resolution on brain tissue, it is important to acknowledge the limitation on the precision with which tDCS is able to target specific areas of the brain. A main issue is the difference between the electrodes used in animals and those used in clinical applications, preventing direct comparisons of current density and voltage distributions between experimental models (higher current densities are often reported in animals). Because in humans the outcome of stimulation depends of the amount of current delivered, it would be necessary to test whether similar dose-response curves occur in animals and attempt stimulation parameters more closely related to clinical studies. Another limiting factor is the considerable protocol variations particularly among animal models. This lack of standardization is deleterious and could contribute to the discrepancy sometimes observed in the literature. The standardization of physical parameters, namely the current density and shape, electrodes size, shape and localization (2 epicranial electrodes vs. 1 epicranial/ 1 outside the skull), the duration and number of stimulations, and the state of animals during the stimulation (awake or anesthetized) would greatly aid in the elucidation of the mechanisms and efficacy of tDCS.

Another often overlooked point is the population on which tDCS is used. The interaction of stimulation polarity, cognitive domain and other intra- and interindividual variables such as anatomic or genetic factors (Plewnia et al., 2013; Kim et al., 2014), personality (Pena-Gomez et al., 2011; Pripfl et al., 2013), cognitive strategy (Berryhill and Jones, 2012) and baseline neuronal activation state (Jacobson et al., 2012) need to be taken into consideration. Likewise, the age at which electrical stimulation occurs (Kessler et al., 2013) might be a key determinants for the physiological and behavioral outcomes of the stimulation. tDCS effects might for example be stronger/different and possibly harmful if applied to the brain during a critical stage of development such as during adolescence when the prefrontal cortex is still not fully mature.

Finally, further basic research is needed to elucidate the duration of the effects of tDCS on memory, which require evaluations at different time-points. The eventual necessity to re-stimulate the brain to maintain the beneficial effects of tDCS has yet to be investigated.

In conclusion, the data reported here are very promising and show that electrical stimulation of the brain is able to improve cognition in humans, in both healthy and in patients with psychiatric or neurological disorders. However, before it can be applied as a therapeutic tool, there is a clear need for method standardization and for a better understanding of its mode of action through the combined use of clinical research and animal models.

### Conflict of interest statement

The authors declare that the research was conducted in the absence of any commercial or financial relationships that could be construed as a potential conflict of interest.
